# Notch Regulation of Hematopoiesis, Endothelial Precursor Cells, and Blood Vessel Formation: Orchestrating the Vasculature

**DOI:** 10.1155/2012/805602

**Published:** 2012-02-23

**Authors:** Vincenza Caolo, Daniel G. M. Molin, Mark J. Post

**Affiliations:** Department of Physiology, Cardiovascular Research Institute Maastricht (CARIM), Maastricht University, Universiteitssingel 50, 6229 ER Maastricht, The Netherlands

## Abstract

The development of the vascular system begins with the formation of hemangioblastic cells, hemangioblasts, which organize in blood islands in the yolk sac. The hemangioblasts differentiate into hematopoietic and angioblastic cells. Subsequently, the hematopoietic line will generate blood cells, whereas the angioblastic cells will give rise to vascular endothelial cells (ECs). In response to specific molecular and hemodynamic stimuli, ECs will acquire either arterial or venous identity. Recruitment towards the endothelial tubes and subsequent differentiation of pericyte and/or vascular smooth muscle cells (vSMCs) takes place and the mature vessel is formed. The Notch signaling pathway is required for determining the arterial program of both endothelial and smooth muscle cells; however, it is simultaneously involved in the generation of hematopoietic stem cells (HSCs), which will give rise to hematopoietic cells. Notch signaling also regulates the function of endothelial progenitor cells (EPCs), which are bone-marrow-derived cells able to differentiate into ECs and which could be considered the adult correlate of the angioblast. In addition, Notch signaling has been reported to control sprouting angiogenesis during blood vessels formation in the adult. In this paper we discuss the physiological role of Notch in vascular development, providing an overview on the involvement of Notch in vascular biology from hematopoietic stem cell to adaptive neovascularization in the adult.

## 1. Introduction

During embryogenesis, the earliest stages of vascular development occur when hematopoietic cell and ECs precursors, hemangioblasts, migrate and differentiate into blood islands. Once hemangioblasts organize in blood islands, they fuse to form the primitive capillary plexus in a process termed vasculogenesis [1, 2]. The cells surrounding the islands will subsequently differentiate into ECs, while those in the centre will form hematopoietic precursors.

The newly formed plexuses grow as a result of angiogenesis, that is, vascular sprouting and tube formation by single ECs within a preexisting capillary plexus, or by intussusceptions, that is, a longitudinal division of existing vessels, involving reorganization of the interendothelial cell junctions, central perforation of the bilayer, followed by interstitial pillar core formation [3, 4]. Subsequently, endothelial cells become surrounded by pericytes and myofibroblasts the newly forming vessel is stabilized into an arteriole (arteriogenesis).

During the process of arteriogenesis, multiple layers of pericytes or SMCs to, respectively, generate small or large vessels of the vascular system cover the formed channel of endothelial cells. A large number of intercellular signaling pathways are implicated in these processes. The analysis of different mouse embryos with targeted mutation of Notch revealed the importance of Notch in all these processes of vascular development [2].

This paper assesses the current knowledge of Notch function in controlling cell fate during vascular development. In particular, we discuss the role of Notch in the formation of hematopoietic cells in the embryo and HSCs self-renewal in the adult. We also review the potential effect of Notch in EPCs activity and its implication for neovascularization. Finally, we report on the function of Notch in sprouting angiogenesis and arterial cell fate specification of both endothelial and smooth muscle cells during the formation of new blood vessels.

## 2. Notch Signaling Pathway and Vascular Development

**Figure 1:**
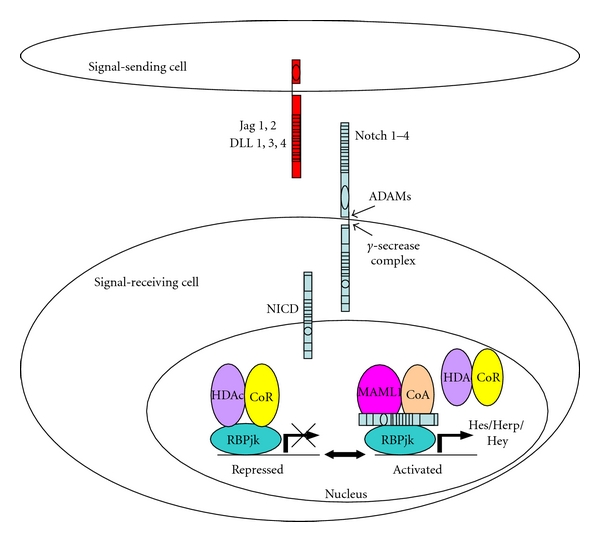
Mammalian cells are equipped with 4 Notch receptors (Notch1-4) and five ligands (Jag1,2 and Dll1,3,4). Notch signaling is triggered upon receptor-ligand interaction, which induces two sequential proteolytic cleavages. The first cleavage, in the extracellular domain, is catalyzed by ADAM metalloproteinases, and the second, within the membrane domain, is facilitated by the *γ*-secretase complex. This second cleavage allows the release and translocation of the Notch intracellular domain (NICD) to the nucleus. Binding of NICD to cotranscription factor RBPjk leads to transcriptional activation of the Notch target genes HES and HERP/HEY by displacement of a corepressor (CoR) and recruitment of the coactivator, mastermind-like protein (MAML1).

The Notch pathway is an evolutionary highly conserved signaling system. Four different Notch receptors, Notch-1 to -4, and five ligands, Delta-like (Dll)-1, -3, -4, and Jagged (JAG)-1, -2, have been identified in vertebrates. The Notch members are single-pass transmembrane protein. Notch receptors are synthesized as single-chain precursors that, after glycosylation by protein O-fucosyl transferase (POFUT1) in the endoplasmic reticulum, are processed into noncovalently linked Notch extracellular (NECD) and intracellular (NICD) domains in the trans-Golgi [5–7]. The receptor-ligand interaction induces two proteolytic cleavages of the receptor. The first is mediated by extracellular proteases, known as A disintegrin and metalloprotease (ADAM), TNF-*α* converting enzyme (TACE) or kuzbanian. Subsequently, the *γ*-secretase complex mediates a second proteolytic cleavage that releases the Notch intracellular domain (NICD) from the membrane. Next, the NICD translocates to the nucleus, where it associates with the DNA binding protein RBP-Jk (also named CSL after mammal CBF1, Drosophila Su(H), and Caenorhabditis elegans LAG-1) and its coactivator Mastermind (Mam) to initiate the transcription of its downstream targets, such as the basic helix-loop-helix proteins hairy/enhancer of split (HES) and hairy-related transcription factors (HRT, HERP, HEY) [7–11], which in turn regulate the transcription of downstream genes (Figure 1).

The importance of Notch in vascular development has been addressed in vivo by gene targeting and mutation studies. Knockout mice of several components of the Notch pathway, such as Notch1, Notch1 plus Notch4, and Jagged1 all resulted in embryonic lethality related to vascular defects [12, 13]. Mice homozygous for a single-point mutation at the intramembranous processing site of Notch1 resembles the null Notch1 phenotype, showing that Notch1 processing is essential for embryonic viability and proper vascular morphogenesis [14].

Both Delta-like 1 (Dll1) deficient and Notch2-hypomorphic mice embryos presented with hemorrhage resulting from poor development of vascular vessels [15, 16]. Also Dll4^+/−^ heterozygous mice exhibit embryonic lethal haploinsufficiency because of vascular remodeling defects [17–19]. In human, the congenital cardiac malformation Alagille syndrome, an autosomal dominant disorder characterized by developmental abnormalities, is caused by mutations of Notch ligand JAGGED1 [20]. Another human disease, CADASIL [21] (cerebral autosomal dominant arteriopathy with subcortical infarct and leukoencephalopathy) is related to a mutation of the Notch3 receptor [22] and affects vascular smooth muscle cell (VSMC) development.

## 3. Notch Signaling Pathway in Hematopoiesis and HSC Self-Renewal

During embryogenesis, first the blood cells originate extra embryonically within the embryonic yolk sac and later are produced intra embryonically in the para-aortic splanchnopleura (P-sP) and aorta-gonad mesonephros (AGM). Subsequently, hematopoiesis takes place in the liver to finally shift towards the spleen and bone marrow [23].

The analysis of mutant mice revealed that Notch signaling is required during hematopoiesis. Hematopoietic cell development was dramatically impaired in Notch1^−/−^ embryos, in which no HSC activity in P-sP and yolk sac was detected. Although the mechanism of action is still poorly understood, these observations unquestionably establish a role for Notch1 in hematopoiesis [24]. In line with these results, Notch cotranscription factor RBPjk mutant embryos showed loss of hematopoietic precursors [24, 25] and absence of GATA2, the transcription factor which is required to maintain the hematopoietic precursor in an undifferentiated state [26]. GATA2 expression in the AGM hematopoietic precursor appears to be regulated by Notch1 signaling through the ligand Jagged1 and an RBPjk dependent pathway. Consistently, mutant Jagged1 embryos were unable to generated hematopoietic cells in the AGM [27].

In the adult, HSCs are mainly quiescent, however in response to stress stimuli, they quickly regenerate damaged hematopoietic cells through a process referred to as HSCs self-renewal. Although Notch signaling together with Wnt signaling has been indicated as a possible regulator of this process [28], its role still remains controversial. Whereas loss of function data do not support any role for Notch in adult HSC, as shown by lack of effects on HSCs after conditional deletion of Notch1, Notch2, Jagged1 genes, or specific expression of a dominant negative mastermind in adult bone marrow cells [29–31], gain of function data suggest a role for Notch in HSCs self-renewal. Forced expression of Notch1ICD in early c-Kit+/Sca1/+Lin- hematopoietic cells (KLS) leads to immortalization of these cells [32]. Ectopic expression of Notch1IC also expanded the number of bone marrow cells promoting their lymphoid differentiation over myeloid lineage commitment [33] similar to Notch4IC overexpression in Lin-cord blood cells [34].

Intravenous injection of cells expressing human Jagged1 into immune deficient nonobese diabetic NOD/SCID mice showed that Jagged1 was able to expand stem cells with pluripotent capacity [35]. Similarly Varnum-Finney et al. [36] have shown that stimulation of bone marrow precursor with a Delta1 fusion protein resulted in inhibition of myeloid differentiation while strongly increased the number of precursor cells. In line with these results, retrovirus-mediated transduction of CD34-KSL cells with Notch target gene Hes1 preserves the long-term proliferation activity of these cells [37]. Conversely, Notch inhibition by a dominant RBPjk/CSL led to accelerated differentiation of HSCs in vitro, indicating that Notch is responsible for maintaining HSCs in an undifferentiated state [28].

## 4. Notch Signaling Pathway and EPCs Activity

Although it is still a matter for debate, growing evidence indicates that precursors of endothelial cells, EPCs, are responsible for postnatal vascularization.

Asahara et al. [38] have isolated EPCs from bone marrow (BM) and have shown that these cells play a role in inducing vessel formation under physiological and pathological conditions. EPCs are believed to reside in the BM together with hematopoietic and hemangioblastic stem cells. EPCs mobilization from BM would occur in response to ischemic stimuli. Their subsequent recruitment at ischemic sites will contribute to the formation of new blood vessels. In the BM there are special microenvironments, so-called niches [39]. Osteoblasts, stromal cells, and ECs populate these niches together with HSCs [40]. These niche cells express Notch ligands such as Dll1 and Jagged1 [41–43]. The interaction between osteoblasts presenting Notch ligands and HSCs expressing Notch receptors is a key molecular mechanism in the regulation of HSCs function in BM niche [36]. Thus, it is possible that a similar mechanism would regulate the function and recruitment of EPCs that reside in this niche.

Consistently, recent studies have shown a role for Notch in EPC activity and postnatal vasculogenesis. Inhibition of Notch has a negative effect on revascularization via impairment of EPC proliferation, differentiation, and mobilization from BM [44]. Particularly, Notch ligand Jagged1 appears to determine the differentiation of BM cells into EPCs. Loss of Jagged1, but not of Dll1, resulted in reduced expression of endothelial genes in BM cells and lower proliferative, migratory and survival ability of BM-EPC-derived cells [44]. Consequently, Jagged1 deficient BM cells showed reduced capacity for vascular regeneration in ischemic tissue. Further research on Notch and its role in EPCs commitment, proliferation, and mobilization is required to fully understand its importance during postnatal vasculogenesis.

## 5. Notch Signaling Pathway during Endothelial Sprouting Angiogenesis

Sprouting angiogenesis is an indispensable process during growth of newly forming blood vessels. Sprouting is initiated by the so-called tip cells that leave the confinement of the basal membrane and starts to spread filopodia into the environment, thereby leading the way for the formation of a new sprout. These tip cells are followed by stalk cells. In this process, the role of Notch signaling is intertwined with another essential regulator of vascular development: vascular endothelial growth factor (VEGF) [45, 46]. During sprouting angiogenesis, tip cells react to a VEGF gradient [47, 48] by migrating and extending filopodia. This response is highly localized as the stalk cells—that follow the tip cell—already respond differently to VEGF. Several recent studies have shown the important role of Notch signaling in regulating the formation and function of these tip cells. Endothelial tip cells express high levels of VEGFR-2. In response to VEGF stimulation, Dll4 expression is upregulated in these cells. The selected tip cell expressing Dll4 interacts with neighboring cells, thereby activating Notch signaling, which in turn prevents migration and filopodia extension that will functionally define these neighboring cells as stalk cells. Thus, in the absence of Notch signaling, endothelial cells continue to form sprouts in response to VEGF, resulting in more sprouts and branches per blood vessel. This indeed has been observed in different model systems such as mouse retina and hindbrain [17, 49–51], zebrafish embryos [52, 53], and xenograft tumor models [51, 54, 55]. Consistently, Dll4^+/−^ mice [17, 49, 50] have serious vascular defects in the retinal vasculature with excessive capillary density, diameter, and filopodia extension of endothelial cells. Similar defects have been observed with Notch inhibition, either in an inducible Notch1 knockout [49], pharmacological blockade by anti-Dll4 antibodies [50, 51], or *γ*-secretase inhibitors [17, 49]. Stalk cells that are unchecked by Dll4 also remained, highly proliferative, as shown in Dll4 or RBP-Jk morphant zebrafish embryos [53]. 

The functional relationship between Notch signaling and VEGF is clear. VEGF induces expression of Dll4 and Notch signaling is required as well as sufficient for this effect. Overexpression of NICD-4 and NICD-1 effectively transactivated deletional fragments of the Dll4 promoter that contained several RBP-Jk binding sites [56]. As ADAMs metalloproteinase can mediate Notch signaling activation even in the absence of typical Notch ligands [57], it is possible that VEGFA-induced ADAM activity results in enhanced Notch signaling, with subsequent increase of Dll4 expression, and shut down of VEGF signaling by VEGFR-2 shedding. Accordingly, VEGF-induced Notch signaling and Dll4 expression was prevented by ADAM blockers [56].

This two way relationship between VEGF and ADAM activity could explain the spatial and/or temporal regulation that causes the “salt and pepper” pattern of Dll4 expression and associated tip and stalk cell distribution described for the mouse retina model [49, 58]. Computational models, supported by genetic mosaic sprouting assay such as time laps microscopy analysis of chimaeric embryoid bodies, have been used to allow various combinations of ECs heterozygous for VEGFR1 or VEGFR2 to compete with wild-type cells for the tip position [59–61]. Cells heterozygous for VEGFR2 showed poor contribution to the tip cell population and predominantly became stalk cells, whereas cells heterozygous for VEGFR1 dominated the tip cell population. Thus, the balance of VEGFR2 and VEGFR1 expression in individual endothelial cells affects their potential to become tips cell during sprouting angiogenesis and this process is mediated by Notch. In response to VEGF, cells which are supposed to have high VEGFR-2 signal will upregulate Dll4 and adopt tip cell position to initiate sprouting [59], possibly in the initial absence of Notch ligands [57] Dll4 in turn triggers its own expression in a positive feed forward fashion. Activated Notch downregulates VEGF signaling, that might be the stop signal [62] to reduce sprouting in the surrounding cells.

Although VEGFA, as the foremost VEGF family member, has been indicated as major player in regulating sprouting angiogenesis through VEGFR2 and upstream of Notch, a recent study has suggested a role for VEGFC/VEGFR3 axis in regulating this process as well [63]. VEGFR3 appears to function in a bimodal fashion. VEGFC expressed by macrophages activates VEGFR3 in tip cells. Activated VEGFR3 in tip cells subsequently contributes to sprouting by activating the MAPK intracellular pathway. Concomitantly, VEGFR3 leads to Notch activation ligand independent. Induced Notch signaling will decrease the sensitivity of tip cells to a VEGF gradient, participating to the conversion of tip cell to stalk cell phenotype at fusion points of vessel sprouts and allowing the growth of the newly forming vasculature.

## 6. Notch Signaling Pathway in Arterial ECs Specification

Whereas Notch maintains HSCs in an undifferentiated state, its activation in the endothelium induces arterial/venous differentiation. The importance of the Notch signaling pathway in regulating arterial differentiation was initially shown in Zebrafish [64, 65] and later confirmed in transgenic mouse models [12, 19, 66–69] and in human endothelial cells [70].

In zebrafish embryo, Notch signaling deficiency resulted in a poorly formed dorsal aorta and posterior cardinal vein. Arterial marker expression, such as EphrinB2, was low and venous markers were ectopically expressed in the arterial compartment. This expression pattern was similar to models with reduced VEGFA signaling, whereas injection of VEGFA mRNA gave a mirror image of expression of the arterial marker EphrinB2 in the posterior cardinal vein [64]. Moreover, induced expression of Notch1 in VEGFA-deficient embryos rescued the expression of EphrinB2. In contrast, VEGFA supplying into Notch signaling deficient embryos was unable to restore arterial differentiation [64].

In human cells, close interaction of VEGF and Notch signaling in arterial differentiation has also been demonstrated. Liu et al. [70] showed that Notch1 receptor and Dll4 ligand are induced by VEGFA in human arterial but not in venous endothelial cells. VEGF-A-induced Dll4, Notch4, EphrinB2 and downregulation of venous markers COUP-TFII and EphB4 expression was also described for mouse embryonic [71] and human bone-marrow-derived mesenchymal stem cells [72]. Also ablation of the venous marker and repressor of Notch signaling COUP-TFII in endothelial cells enabled veins to express Notch signaling components, acquiring an arterial phenotype, whereas COUP-TFII ectopic expression resulted in fusion of arteries and veins (AV-shunts) in transgenic mouse embryos [73].

Occasionally surviving Dll4 heterozygous mice displayed a lack of arterial markers in their vasculature, as do other Notch signaling deficient mice such as the RBP-Jk and Hey1 and Hey double-mutants. Studies in cultured endothelial cells confirmed that Dll4-induced Notch signaling upregulates EphrinB2 expression [74]. Furthermore, the overexpression of activated Notch4 (NICD-4) in adult mice was also able to induce the expression of EphrinB2 in the venous compartment. These mice were characterized by arteriovenous malformation that was reversible after repression of Notch4 expression [75].

These studies provide evidence that both Notch and VEGF are involved in a signaling cascade that is essential for arterial-venous differentiation in which Notch signaling acts downstream of the VEGF pathway.

## 7. Notch Signaling Pathway in vSMC Differentiation

Despite the common origin of hematopoietic cells and endothelial cells, vascular SMCs come from a different embryonic source. Lineage mapping studies have identified at least eight independent possible SMC progenitors such as neuronal crest, pericardium, mesothelium, secondary heart field, somites, mesoangioblasts, stem and progenitor cells, microvascular SMCs, and perycites [76]. Intriguingly, although all these progenitors have different origin and molecular characteristics, they all have the ability to differentiate in a cell type, which will express SMC marker genes such as *α*SMActin (*α*SMA), SM-MHC (Smooth Muscle Myosin Heavy Chain), SM22*α*, and SM-calponin. Subsequently, these SMCs differentiate further taking part to the formation of either arterial or venous vessels.

The Notch signaling pathway plays a role in controlling SMC fate specification, although debates about the precise role are still ongoing. Besides affecting arterial ECs differentiation, Notch signaling has been reported to specify arterial-venous identity of vascular SMCs as well [77]. Among the receptors, Notch3 is expressed in vascular SMCs from arteries but not from veins [77]. Notch3^−/−^ mice showed serious arterial defects, such as enlarged arteries with a thinner layer of SMCs [77] that were poorly differentiated [77, 78] compared to wild-type animals. This is consistent with in vitro data showing that ligand induced NOTCH signaling upregulated the expression *α*SMA and SM-MHC promoting VSMC differentiation [79, 80] via RBP-Jk. Among the ligands, endothelial Jag1 appears to be essential in regulating vascular morphogenesis by inducing Notch signaling in the neighboring SMC. Endothelial-specific deletion of Jag1 causes serious cardiovascular abnormalities and embryonic lethality in mice [81]. Surprisingly, these embryos differ from embryos in which general Notch signaling has been abrogated as they still present with normal Notch activation and arterial differentiation of the endothelium. This suggests that endothelial Jag1 mainly acts by inducing the differentiation of the adjacent SMCs [81]. Notch3 activation by endothelial Jag1 is further supported by enhanced expression of Notch3 in SMC cocultured with Jag1 expressing endothelial cells, suggesting a positive feedback loop. Indeed, Liu et al. [80] demonstrated that activated Notch3 signaling initiates a positive feedback loop that promotes its own expression and propagates Notch signaling to the other SMCs, meanwhile inducing SMC differentiation markers, such as *α*SMA, CALPONIN, and SM-MHC. These data are in contrast with other studies in which Notch target genes have been reported to antagonize SRF (serum response factor) and myocardin, the main transcription regulators of VSMC gene expression and SMC differentiation [82, 83]. The controversy between these reports might relate to the balance between Notch ICD that induces the expression of VSMC markers and the concomitant expression of Notch target genes such as Hey1 and Hey2 that turn off these markers as part of a negative feedback loop. The validity of this model has been experimentally addressed by using ECs and SMC co-cultures [83, 84]. Hey1 and Hey2 were indeed able to repress *α*SMA expression and to antagonize Notch-induced *α*SMA expression in vitro by decreasing Notch ICD/RBP-Jk binding and transactivation of the *α*SMA promoter.

## 8. Conclusions

In this paper, we report on the essential role of the Notch signaling pathway during embryonic vascular development and postnatal neovascularization, with special focus on its ability to affect fate specification of different cell types.

**Figure 2:**
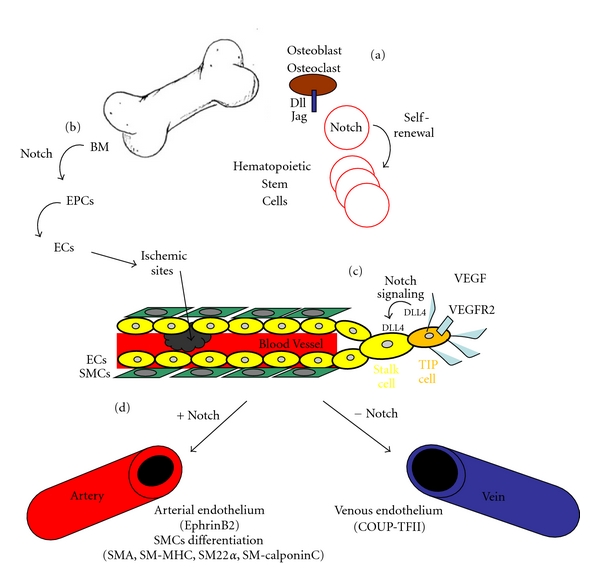
Scheme showing the essential functions of Notch in blood and vascular cell types (a) Osteocyte Dll/Jag-induced Notch signaling in hematopoietic stem cells (HSCs) is involved in their generation and self-renewal in the adult. (b) Notch signaling pathway is required for endothelial progenitor cells (EPCs) development and function. EPCs from bone marrow niches will, in response to Notch, differentiate in endothelial cells (ECs) and populate sites of ischemia participating in vascularization. (c) Schematic representation of a growing blood vessel. In angiogenesis, Notch ligand DLL4 is upregulated in response to VEGF/VEGFR2 signaling in tip cells, that are specified ECs (dark yellow) capable of sprouting by extending filopodia. Upregulated DLL4 in tip cells activates Notch in the neighbor stalk ECs (light yellow) inhibiting their sprouting capacity. Notch activation in stalk cells will result in further DLL4 upregulation, which eventually will activate Notch in adjacent cells. SMCs, covering the endothelium are represented in green. (d) Notch induces arterial specification of ECs by upregulating the expression of arterial markers, such as EphrinB2. The lack of Notch in venous endothelial cells allows the expression of venous markers, such as COUP-TFII. Notch regulates also smooth muscle cell (SMC) differentiation by inducing the expression of SMC-specific markers (e.g., *α*SMA, SM-MHC, SM*α*22, and SM calponin).

Notch signaling appears to be indispensable early during vasculogenesis by affecting hematopoietic cell development. This function is preserved in the adult during HSCs renewal, and contributes to regenerate damaged hematopoietic cells in response to stress stimuli (Figure 2(a)). Recently, a role for Notch in controlling mobilization and differentiation of EPCs from the BM niches has been reported. EPCs represent an important therapeutic potential for vascular regeneration, as they are able, in response to specific stimuli, to reach ischemic sites and regenerate blood vessels (Figure 2(b)). A more detailed understanding of Notch signaling mechanism would certainly help to improve EPCs mediated neovascularization following ischemia. Besides affecting blood cells generation and renewal, Notch signaling is implicated in the differentiation of blood vessel cells such as ECs and SMCs. Activation of Notch signaling is required for controlling sprouting angiogenesis of ECs, as its absence will result in excessive sprouting (Figure 2(c)). As a result, the newly formed vascular network is not functional. Its role in inducing arterial differentiation of endothelial and smooth muscle cells has also received large attention. Notch activation determines whether a blood vessel will become an artery or a vein, by inducing arterial and repressing venous marker expression, respectively (Figure 2(d)).

Notch's extraordinary ability to regulate specification of different cell types within the vascular system makes it a powerful therapeutic tool to target both pathological and physiological vascularization.
